# Progress Toward Poliomyelitis Eradication — Pakistan, January 2023–June 2024

**DOI:** 10.15585/mmwr.mm7336a2

**Published:** 2024-09-12

**Authors:** Chukwuma Mbaeyi, Anwaar ul Haq, Rana Muhammad Safdar, Zainul Khan, Melissa Corkum, Elizabeth Henderson, Zubair M. Wadood, Muhammad Masroor Alam, Richard Franka

**Affiliations:** ^1^Global Immunization Division, Center for Global Health, CDC; ^2^National Emergency Operation Center, Islamabad, Pakistan; ^3^World Health Organization, Islamabad, Pakistan; ^4^UNICEF, Islamabad, Pakistan; ^5^Division of Viral Diseases, National Center for Immunization and Respiratory Diseases, CDC; ^6^World Health Organization, Polio Eradication Department, Geneva, Switzerland; ^7^World Health Organization, Amman, Jordan.

**Summary**

**What is already known about this topic?**

Wild poliovirus type 1 (WPV1), the only circulating wild poliovirus serotype, remains endemic in only two countries, Afghanistan and Pakistan, which share borders and are considered a single epidemiologic block.

**What is added by this report?**

During January 2023–June 2024, 14 WPV1 cases were reported in Pakistan, compared with 21 WPV1 cases during January 2022–June 2023. However, widespread transmission of poliovirus reemerged in the historical polio reservoirs of Karachi, Peshawar, and Quetta, as evidenced by a spike in sewage samples testing positive for WPV1.

**What are the implications for public health practice?**

Addressing community demands for essential services along with redoubling efforts to track and vaccinate children who are repeatedly missed during polio vaccination activities will help to bring the goal of WPV1 elimination within reach for the Pakistan polio program.

## Abstract

Since its launch in 1988, the Global Polio Eradication Initiative has made substantial progress toward the eradication of wild poliovirus (WPV), including eradicating two of the three serotypes, and reducing the countries with ongoing endemic transmission of WPV type 1 (WPV1) to just Afghanistan and Pakistan. Both countries are considered a single epidemiologic block. Despite the occurrence of only a single confirmed WPV1 case during the first half of 2023, Pakistan experienced widespread circulation of WPV1 over the subsequent 12 months, specifically in the historical reservoirs of the cities of Karachi, Peshawar, and Quetta. As of June 30, 2024, eight WPV1 cases had been reported in Pakistan in 2024, compared with six reported during all of 2023. These cases, along with more than 300 WPV1-positive environmental surveillance (sewage) samples reported during 2023–2024, indicate that Pakistan is not on track to interrupt WPV1 transmission. The country's complex sociopolitical and security environment continues to pose formidable challenges to poliovirus elimination. To interrupt WPV1 transmission, sustained political commitment to polio eradication, including increased accountability at all levels, would be vital for the polio program. Efforts to systematically track and vaccinate children who are continually missed during polio vaccination activities should be enhanced by better addressing operational issues and the underlying reasons for community resistance to vaccination and vaccine hesitancy.

## Introduction

Although the Global Polio Eradication Initiative (GPEI) has achieved substantial progress since its establishment in 1988, the goal of polio eradication has remained elusive. Indigenous wild poliovirus type 1 (WPV1) circulation has never been interrupted in Pakistan, and endemic circulation also continues in Afghanistan (*1*,*2*). Both countries constitute a single epidemiologic block because of substantial cross-border population movements along their respective northern and southern borders (*3*). The 2022–2026 GPEI Strategic Plan targeted ending all transmission in 2023 (*4*); however, WPV1 transmission in both countries has continued into 2024. This report describes Pakistan’s progress toward eliminating indigenous WPV1 transmission during January 2023–June 2024 (*5*,*6*).

## Methods

### Data Sources

Poliovirus surveillance data and vaccination information (campaign reports and routine immunization coverage surveys) as of June 2024 were provided by the Pakistan National Emergency Operations Centre and other GPEI partners, including UNICEF and the World Health Organization (WHO). Weekly poliovirus country and regional surveillance reports, including environmental surveillance data, were also reviewed.

### Analysis

Genomic sequencing and analyses from the Pakistan National Institute of Health poliovirus laboratory determined the genetic relationship among polioviruses identified in specimens collected from patients with WPV1 infection and from environmental sewage samples. This activity was reviewed by CDC, deemed not research, and was conducted consistent with applicable federal law and CDC policy.*

## Results

### Immunization Activities

**Routine immunization.** WHO and UNICEF estimated Pakistan’s national coverage with 3 doses of oral poliovirus vaccine^†^ (OPV) and 1 dose of inactivated poliovirus vaccine (IPV) (containing polio vaccine virus types 1, 2, and 3), by age 12 months at 86% for each vaccine during 2023; Pakistan introduced a second IPV dose in 2021, and 2-dose IPV coverage was estimated at 84% (*7*). A 2021 third-party survey sponsored by Gavi, the Vaccine Alliance (https://www.gavi.org), indicated that the proportion of children aged 12–23 months who had received 3 routine immunization OPV doses ranged by province from 45.1% in Balochistan to 94.9% in Punjab. None of the districts in the provinces of Balochistan, Khyber Pakhtunkhwa, and Sindh achieved ≥80% 3-dose routine immunization OPV coverage, compared with 31 (86%) of 36 districts in Punjab province.

**Supplementary immunization activities.** Since the synchronized withdrawal of trivalent OPV (tOPV; containing Sabin-strain types 1, 2, and 3) by all OPV-using countries in 2016 after the eradication of wild poliovirus (WPV) type 2 (*8*), polio supplementary immunization activities (SIAs)^§^ in Pakistan have primarily been conducted using bivalent OPV (bOPV; containing Sabin-strain types 1 and 3). During 2023, three national immunization day (NID) and seven subnational immunization day (SNID) campaigns were conducted using bOPV. NIDs in Pakistan target 45 million children aged <5 years, whereas SNIDs target smaller populations, depending on the areas identified by ongoing risk assessments. Fractional-dose IPV^¶^ was administered during vaccination activities conducted in six districts of south Khyber Pakhtunkhwa in June 2023, in Khyber and Peshawar districts of Khyber Pakhtunkhwa in August 2023, and in Chaman and Killa Abdullah districts of Balochistan province in October 2023.

To date in 2024, two NIDs (January and February) and two SNIDs (April and June) as well as an outbreak response campaign in March have been conducted in Pakistan. In the seven districts of south Khyber Pakhtunkhwa, an area facing considerable security challenges, as many as 706,613 eligible children aged <5 years were not vaccinated during the January 2024 NID, because SIAs could not be safely carried out in those areas. In Dera Ismail Khan, the district with the largest number of eligible children (372,726 children aged <5 years), campaigns could not be conducted during three of the four November 2023–April 2024 SIAs. The program continues to be hampered by repeated community boycotts during SIAs for reasons mostly unrelated to vaccination, such as requests for clean water and electricity services that are selectively provided by the government. Safety remains an ongoing concern for frontline polio program workers in several priority areas.

Lot quality assurance sampling (LQAS)** surveys, which assess SIA quality, continue to indicate substantial gaps in vaccination campaign quality. Based on a 90% pass threshold and surveyed using finger-marking (the marking of a child’s fingernail with indelible ink by vaccinators as a program indicator of having recently received OPV), the proportion of subdistrict union councils reaching the threshold ranged from 82% in Balochistan province to 89% in Punjab province for the June 2024 SNIDs; however, at the district level, pass rates were as low as 25% in Loralai district and 37.5% in Killa Abdullah district, both in Balochistan province. A total of 599,105 children (3.3% of the target population) were missed during the June 2024 SNIDs, including 51,199 refusals.

### Poliovirus Surveillance

**Acute flaccid paralysis surveillance.** A reported nonpolio acute flaccid paralysis (NPAFP)^††^ rate of ≥2 cases per 100,000 children aged <15 years is the WHO benchmark for surveillance sufficiently sensitive to detect an occurrent case of poliomyelitis. Pakistan reported a national NPAFP rate of 20.2 cases per 100,000 persons aged <15 years in 2023 (Table); provincial rates ranged from 11.6 to 33.1, exceeding the recommended benchmark. As of June 9, 2024, the annualized 2024 national NPAFP rate is 17.4. Stool specimen adequacy^§§^ during 2023 and 2024 exceeded the ≥80% target nationally and in each province, except in Islamabad in 2023 (76.9%). District-level performance indicators continue to indicate gaps in surveillance quality, especially in program priority areas.

**TABLE:** Acute flaccid paralysis surveillance indicators and number of wild poliovirus cases reported, by province and period — Pakistan, January 2023–June 2024

Metric	Reporting period	Region							
		Azad Jammu and Kashmir	Gilgit-Baltistan	Islamabad	Khyber Pakhtunkhwa	Punjab	Balochistan	Sindh	Total
**AFP surveillance indicators**									
No. of AFP cases (NP AFP rate)*	2023	585 (25.9)	172 (21.1)	251 (33.1)	4,934 (24.0)	9,288 (19.8)	759 (11.6)	3,782 (16.5)	**19,771 (20.2)**
	2024^†^	225 (22.6)	72 (20.0)	90 (26.9)	1,856 (20.4)	3,319 (16.1)	324 (11.1)	1,622 (16.1)	**7,508 (17.4)**
**% with adequate stool specimens^§^**	2023	89.2	89	76.9	81.9	84.5	87.1	83.8	**84.1**
	2024	93.3	84.7	82.2	87.2	85.3	87.7	88.8	**86.8**
**No. of reported poliovirus cases**									
**WPV1 cases**	Jan–Jun 2023	0	0	0	1	0	0	0	**1**
	Jul–Dec 2023	0	0	0	3	0	0	2	**5**
	Jan–Jun 2024	0	0	0	0	0	6	2	**8**
	**Total**	**0**	**0**	**0**	**4**	**0**	**6**	**4**	**14**
**cVDPV2 cases**	Jan–Jun 2022	0	0	0	0	0	0	0	**0**
	Jul–Dec 2022	0	0	0	0	0	0	0	**0**
	Jan–Jun 2023	0	0	0	0	0	0	0	**0**
	**Total**	**0**	**0**	**0**	**0**	**0**	**0**	**0**	**0**

**Abbreviations:** AFP = acute flaccid paralysis; cVDPV2 = circulating vaccine-derived poliovirus type 2; NP = nonpolio; WHO = World Health Organization; WPV1 = wild poliovirus type 1.

* NP AFP cases per 100,000 persons aged <15 years.

^†^ Annualized.

^§^ Defined as two stool specimens collected ≥24 hours apart within 14 days of paralysis onset and arriving at a WHO-accredited laboratory with reverse cold chain storage maintained and without leakage or desiccation.

**Environmental surveillance**. A network of 124 environmental surveillance (ES) (the systematic sampling and testing of sewage for the presence of poliovirus) collection sites in Pakistan serves to supplement poliovirus surveillance. Sewage samples collected monthly at these sites are tested for polioviruses and other enteroviruses. During 2023, among 2,563 sewage samples tested, 126 (5%) were positive for WPV1, compared with 37 (3%) of 1,325 in 2022. To date in 2024, among 942 tested sewage samples, 203 (22%) have tested positive for WPV1. As of June 30, 2024, ES samples positive for WPV1 had been identified in Sindh (mostly in Karachi), Balochistan, Islamabad, Khyber Pakhtunkhwa, and Punjab, indicating widespread circulation of the virus in the country. Approximately 60% of all WPV1-positive ES isolates were reported from the traditional polio reservoirs in the cities of Karachi, Peshawar, and Quetta.

**FIGURE 1:**
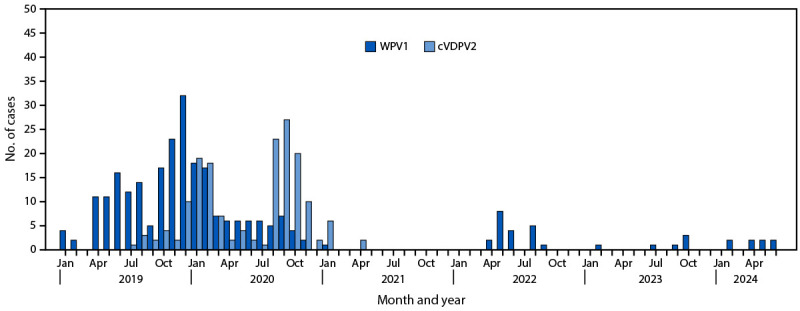
Reported cases of wild poliovirus type 1 and circulating vaccine-derived poliovirus type 2, by month — Pakistan, January 2019–June 2024 **Abbreviations**: cVDPV2 = circulating vaccine-derived poliovirus type 2; WPV1 = wild poliovirus type 1.

**FIGURE 2:**
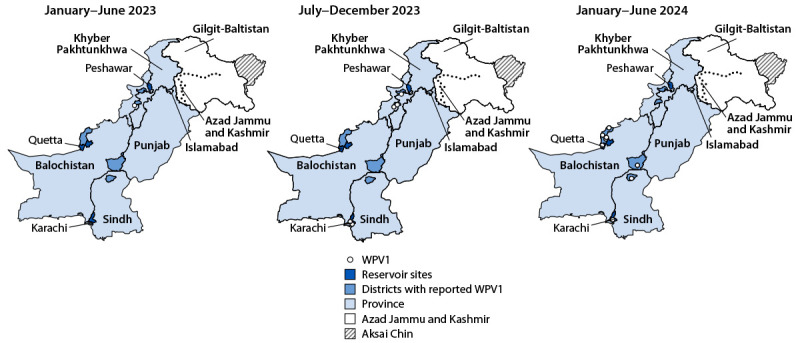
Location of wild poliovirus type 1 cases, by province and period — Pakistan, January 2023–June 2024 **Abbreviation:** WPV1 = wild polio virus type 1.

**Epidemiology of poliovirus cases.** Six WPV1 cases were reported in Pakistan in 2023, compared with 20 cases in 2022, one in 2021, and 84 in 2020 (*5*,*9*) (Figure 1) (Figure 2). As of June 30, 2024, eight WPV1 cases had been reported in 2024, compared with a single case reported during the same period in 2023. Among the six WPV1 cases reported in 2023, three were from Bannu district, Khyber Pakhtunkhwa province, two from Karachi East district, Sindh province, and one from Orakzai district, Khyber Pakhtunkhwa province. Among the eight cases reported to date in 2024, six have been reported from Balochistan province and two from Sindh province. Among the 14 WPV1 cases identified during January 2023–June 2024, patients ranged in age from 9 months to 144 months (median = 30 months); seven patients had never received OPV through routine immunization, two had received 1–2 doses, while the remaining five had received 3 routine immunization OPV doses. No circulating vaccine-derived poliovirus type 2 (cVDPV2)^¶¶^ cases have been reported in Pakistan since April 23, 2021, when the last of 165 cVDPV2 cases that occurred during July 2019–April 2021 was reported (Table) (Figure 1) (Figure 2).

**Genomic sequence analysis of WPV1 isolates**: Analysis of the region coding the VP1 capsid protein of WPVs is used to classify them into genetic clusters (i.e., those that share ≥95% sequence identity). Among the six WPV1 cases and 126 WPV1-positive ES isolates reported in 2023, nine belonged to groups of viruses derived from the YB3C cluster, endemic to Pakistan; the other 123 belonged to groups of viruses derived from the YB3A cluster, cocirculating in eastern Afghanistan. Among the eight WPV1 cases reported to date in 2024, six belonged to the YB3A4A cluster, and two belonged to the YB3A4B cluster. In addition, six orphan viruses (>1.5% VP1 nucleotide divergence, indicating gaps in AFP surveillance) were identified during the preceding 12 months.

## Discussion

Compared with the previous reporting period (January 2022–June 2023), more extensive WPV1 transmission has been evident in Pakistan, beginning in the second half of 2023 through the first half of 2024. The number of reported WPV1 cases rose from a single case in the first half of 2023 to five additional cases during the second half of the year. Eight WPV1 cases had been reported as of June 2024, with cases appearing in historical polio reservoirs in Balochistan and Sindh provinces. This has occurred despite considerable progress in the security-compromised south Khyber Pakhtunkhwa region, which has not reported a WPV1 case in 2024, and the absence of viruses derived from the YB3C genetic cluster of WPV1 from circulation since November 2023. Concomitantly, the proportion of ES samples testing positive for WPV1 has increased from 5% in 2023 to 22% as of June 2024.

The increase in WPV1-positive ES isolates in 2023 and 2024 has been marked by widespread virus transmission in the core polio reservoirs (Karachi, Peshawar, and Quetta blocks) and central Pakistan, even though case counts have remained relatively low. Reasons for this likely include substantial gaps in the quality of surveillance at the district and subdistrict levels, despite AFP surveillance performance indicators remaining high nationally and provincially. The Pakistan polio program continues to work to ameliorate these gaps through regular reviews of its surveillance sites. More attention needs to be placed on ensuring that the proportion of stool specimens collected from persons with AFP meets the 80% benchmark for adequacy to increase the likelihood of isolating WPV1 from AFP cases.

Routine immunization coverage with OPV and IPV has improved in recent years. IPV protects against paralysis; however, because it is an inactivated vaccine that does not replicate in the intestinal tract as does OPV, it does not prevent the spread of poliovirus. This could partly explain the relatively low number of WPV1 cases reported in the context of widespread WPV1 circulation as evidenced by environmental surveillance. However, one half of all WPV1 patients had never received OPV through routine immunization, indicating population immunity gaps. Assessments of SIAs also continue to indicate deficiencies in campaign quality for operational reasons in some areas, necessitating a redoubling of efforts to systematically track and vaccinate children who are repeatedly missed during polio SIAs. Although overall community acceptance of vaccination remains high, critical challenges exist in core reservoirs that require enhanced community engagement. Whenever feasible, vaccination activities need to be synchronized with those of neighboring Afghanistan.

### Limitations

The findings in this report are subject to at least two limitations. First, caregiver recall might distort reported vaccination histories during AFP case investigations. Second, even when a given child’s finger is marked as evidence of vaccination during SIAs, it might not accurately reflect the actual vaccination status of the child, because finger-marking sometimes occurs even when a child has not received the vaccine dose.

### Implications for Public Health Practice

Pakistan’s frontline workers continue to imperil their safety under challenging conditions to eliminate polio from the country. Although transmission is unlikely to be interrupted by the end of 2024, Pakistan maintains a strong political commitment to achieving the goal in the near future (*10*). Addressing community demands for essential services, such as clean water and electricity, could help improve community participation in vaccination activities. This, along with concerted efforts to track and vaccinate repeatedly missed children, will help to bring the goal of eradication within reach for Pakistan and the GPEI.

## Footnotes

* 45 C.F.R. part 46, 21 C.F.R. part 56; 42 U.S.C. Sect. 241(d); 5 U.S.C. Sect. 552a; 44 U.S.C. Sect. 3501 et seq.

† After the 2016 global synchronized withdrawal of type 2 vaccine viruses, bivalent OPV (bOPV, containing vaccine virus types 1 and 3) has been recommended in all OPV-using countries; however, other OPV formulations, including monovalent OPV (mOPV, containing type 2 vaccine virus) are used in certain situations, including response activities. Estimates of OPV coverage are made using a variety of methods and can include different vaccine formulations.

§ SIAs are mass house-to-house vaccination campaigns targeting children aged <5 years with OPV, regardless of the child’s vaccination history.

¶ Fractional-dose inactivated poliovirus vaccine administration is a dose-sparing vaccination strategy that provides intradermal administration of one fifth the full intramuscular IPV dose.

** LQAS uses a small sample size to assess the quality of vaccination activities after SIAs in union councils (referred to as “lots”). LQAS surveys seek evidence of vaccination (finger marking) by randomly selecting 60 children within each lot. If the number of unvaccinated persons in the sample exceeds three, then the union council SIA is classified as having failed at a pass threshold of ≥90%, and additional vaccination activities in those areas are recommended. If the threshold of ≥90% (three or fewer unvaccinated children) is met, the union council SIA is classified as having passed.

†† AFP cases that are discarded as not having laboratory or other proof of poliovirus as the cause are called nonpolio AFP cases.

§§ Stool specimens are considered adequate if two specimens are collected ≥24 hours apart within 14 days of paralysis onset and arrive at a WHO-accredited laboratory with reverse cold chain storage maintained and without leakage or desiccation. The standard WHO stool specimen indicator target is adequate stool specimen collection from ≥80% of AFP cases.

¶¶ cVDPVs emerge as the result of regaining of neurovirulence of attenuated OPV viruses after prolonged circulation in underimmunized populations; cVDPVs can lead to paralysis.
